# Osteopontin is involved in TLR4 pathway contributing to ovarian cancer cell proliferation and metastasis

**DOI:** 10.18632/oncotarget.21844

**Published:** 2017-10-12

**Authors:** Cong Xu, Hua Li, Miao Yin, Tao Yang, Liguo An, Guiwen Yang

**Affiliations:** ^1^ Shandong Provincial Key Laboratory of Animal Resistance Biology, College of Life Sciences, Shandong Normal University, Jinan 250014, China; ^2^ College of Pharmacy, Binzhou Medical University, Yantai 264003, China

**Keywords:** osteopontin, TLR4, proliferation, metastasis, ovarian cancer

## Abstract

Tumor cell proliferation and metastasis are critical for tumor progression and lead to death of cancer patients. TLR4 is a member of the toll-like receptor (TLR) family, which promotes tumor growth, metastasis and immune escape. Osteopontin (OPN), a phosphorylated glycoprotein extensively expressed in multiple cell-types, plays important roles in tumorigenesis, metastasis and infiltration, and participates in signal transduction of innate immunity. However, it is unclear whether TLR4 has any relationship with OPN. The current study investigated the role of TLR4 and OPN in tumor proliferation and metastasis, and the potential effect of TLR4 signaling on OPN using the human ovarian cancer cell line HO-8910PM. High expression levels of TLR4 and OPN were detected in HO-8910PM cells, which promoted the proliferation, migration and invasion of tumor cells. Lipopolysaccharide (LPS) induced activation of TLR4 up-regulated OPN, increasing the malignant phenotype of cells. RNAi-mediated knockdown of OPN reduced significantly the metastatic phenotype activated by TLR4. Taken together, our study demonstrates that OPN contributes to the ovarian cancer cell proliferation and metastasis, which is activated by TLR4 signaling pathway. It provides new insights for the mechanisms of tumor development and metastasis, and suggests targeting TLR4 and OPN as an intervention in the ovarian cancer treatment.

## INTRODUCTION

Toll-like receptors (TLRs) are well conserved pattern-recognition receptors (PRRs) that are primarily expressed in human immune cells and epithelial cells. These receptors play important roles in innate immune and adaptive immune responses through recognition of pathogen-associated molecular patterns (PAMPs) [1, 2]. Recent studies have reported that TLRs are also expressed in many tumor cells, and play key functions in tumorigenesis, development, and metastasis [3, 4]. Toll-like receptor 4 (TLR4) is a member of the TLR family which is well-known for recognizing lipopolysaccharide (LPS), a component in many Gram-negative bacteria and selective Gram-positive bacteria [5]. TLR4 was reported to promote tumor growth, metastasis, and immune escape in many tumor cells [6, 7]. Haricharan *et al*. demonstrated that TLR4 activated secretion of pro-growth cytokines that promoted the growth of TP53 mutant breast cancer cells [8]. Kim *et al*. found that in epithelial ovarian carcinoma cells expressing TLR4 and MyD88, pro-inflammatory cytokines were constitutively secreted and directly contributed to cancer cell survival and progression [9]. He *et al*. used LPS to activate the TLR4 signal in lung cancer cells, which induced the expression of immune suppression factors, such as TGF-β, VEGF, IL-8, and assisted the immune escape of cancer cells [10].

Osteopontin (OPN) is a phosphorylated glycoprotein that is ubiquitously expressed in multiple tissues and cell-types. It has been shown to have multiple functions, such as regulation of macrophage homing, activation of T cells, neovascularization, inhibition of apoptosis, and reconstruction of extracellular matrix [11, 12]. OPN also plays important roles in the formation, metastasis and invasion of malignant tumor cells [13, 14]. Several studies have proven that increased OPN expression is associated with advanced tumor stage, poor prognosis and tumor metastasis [15, 16]. Overexpression of OPN increased cell proliferation, migration, invasion and tumor formation in human ovarian, prostate, lung and liver cancer cells [17–19]. In addition, high levels of OPN expression were observed in multiple tumor tissues and blood samples of patients with malignant tumors, indicating that OPN overexpression contributed to cancer development and metastasis [20, 21].

Moreover, Zhao *et al*. demonstrated that the intracellular OPN expression could be increased by activating TLR4 in mouse macrophages [22]. These results suggest a possible relationship between TLR4 and OPN in immune cells. As OPN is involved in innate immunity signal transduction [23, 24], it is likely to be involved in signals mediated by TLR4. Therefore, we seek to investigate whether TLR4 and OPN work together to mediate tumor cell invasion and metastasis.

Ovarian cancer is one of the common cancers in female reproductive organs. It ranks eighth in the incidence of all cancers among women worldwide, and is the leading cause of cancer-related death from gynecological cancers in China [25]. In the current study, the human ovarian cancer cell line, HO-8910PM, was investigated to examine the expression levels of TLR4 and OPN in ovarian cancer cells. We used TLR4 specific agonist and inhibitor, and shRNA-mediated knockdown of OPN to explore the effect and mechanism of TLR4 signaling on OPN expression and the metastatic phenotype of cancer cells. Taken together, our study investigates the mechanisms of tumorigenesis, development, and metastasis, and provides evidence for the combined targeting of TLR4 and OPN in the treatment of ovarian cancer.

## RESULTS

### Expression of TLR4 and OPN in ovarian cancer cells

**Figure 1:**
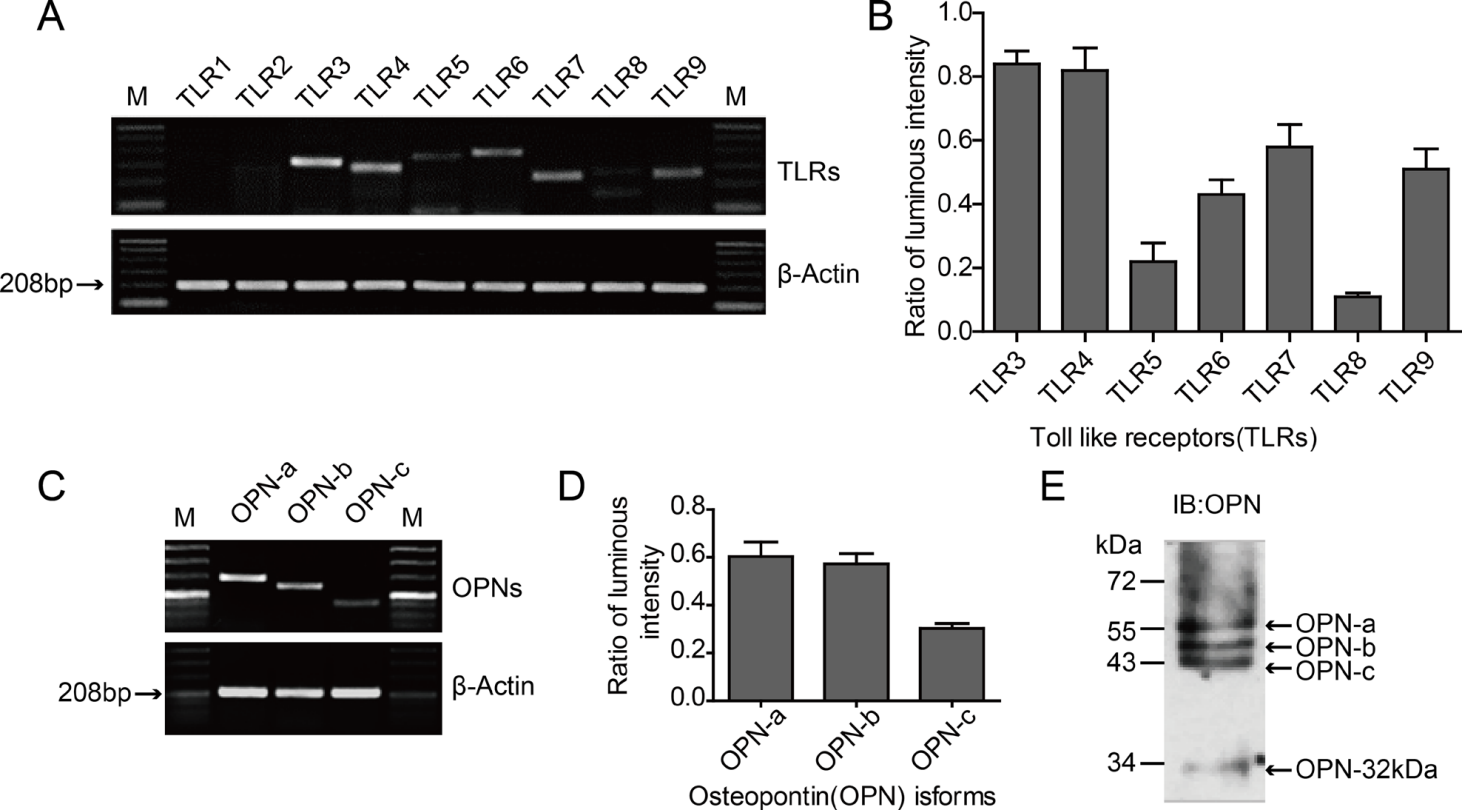
Expression of TLRs and OPN isoforms in HO-8910PM cells (**A**) mRNA expression of various TLRs analyzed by RT-PCR. M: Marker. (**B**) Luminous intensity ratios of TLRs to β-actin. (**C**) mRNA expression of OPN isoforms OPN-a, -b and -c analyzed by RT-PCR. (**D**) Luminous intensity ratios of OPN isoforms to β-actin. (**E**) Expression of OPN protein measured by immunoblotting (IB) on cell supernatants. (A–E) Data are shown as mean ± SD of three independent experiments.

The mRNA expression levels of TLR3-TLR9 were measured in HO-8910PM cells, and quantified using Band Scan5.0 software to analyze the optical density ratio between the TLR gene amplicon and the internal reference amplicon. TLR1 and TLR2 were not detected in the cells, while high levels of TLR3 and TLR4 were detected (Figure 1A–1B).

Three kinds of splicing isoforms are present in OPN: OPN-a (full-length form), OPN-b (lacking exon 5), and OPN-c (lacking exon 4) [26]. Specific primers were designed according to the differences in the mRNA structures of the three isoforms, and the basal expression levels in the tumor cells were tested through RT-PCR using HO-8910PM cell cDNA as the template. The mRNAs of OPN-a, OPN-b, and OPN-c exhibited different expression levels in HO-8910PM cells; while the expression level of OPN-a was similar to that of OPN-b, OPN-c was lower than OPN-a and OPN-b (Figure 1C–1D). The OPN protein levels in cellular supernatant were further tested via immunoblotting (IB), and the results were consistent with RT-PCR (Figure 1E).

The human OPN polyclonal antibody used in IB can identify the C-terminal segment of OPN created by the digestion of thrombin or matrix metalloproteinase. Our results revealed that the OPN segment with a molecular weight of 32 kDa (OPN-32kDa) was expressed in HO-8910PM cells (Figure 1E). This OPN segment contains only the CD44/CD44v-binding domain digested by thrombin or MMP-2/MMP-7 at the hypersensitive site in the SVVYGLR sequence.

### Effect of TLR4 on proliferation and metastatic potential of ovarian cancer cells

**Figure 2:**
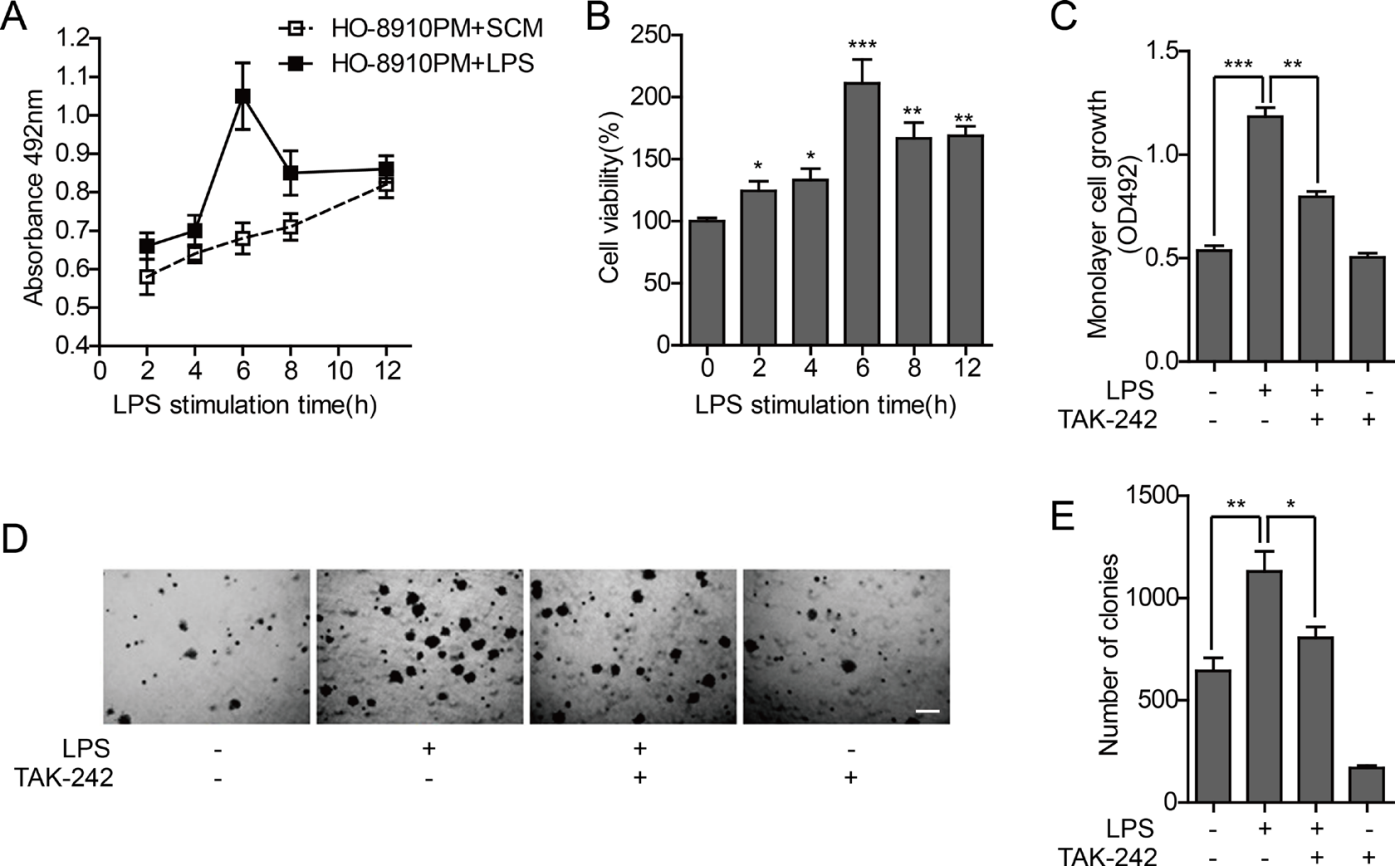
Effect of TLR4 on proliferation ability of HO-8910PM cells (**A**) Monolayer cell growth rates measured by MTT assay in cells treated with serum-containing medium (SCM) or LPS at indicated times. (**B**) Quantification of cell proliferation based on assay in (A). (**C**) Monolayer cell growth rates measured by MTT assay in cells treated with LPS and/or TAK-242. (**D**) Inverted microscopic images of cells treated with LPS and/or TAK-242 in colony formation assay at low magnification (Scale bar: 100 μm). (**E**) Colony numbers were counted using ImageJ software. (A–E) Data are shown as mean ± SD of three independent experiments. ^*^P < 0.05; ^**^P < 0.01; ^***^P < 0.001.

The MTT assay was used to test *in vitro* proliferation activity of tumor cells. Without LPS stimulation, the proliferation activity of tumor cells increased during 12 h. The cell proliferation significantly changed by LPS stimulation, and the maximum absorbance value at 429 nm was present after 6 h, with a proliferation rate of approximately 137.1% compared to cells without stimulation (Figure 2A–2B). To investigate the effect of TLR4 signal block on cell proliferation, the TLR4 inhibitor TAK-242 was used. The LPS-stimulated increase in the proliferation of tumor cells was significantly reduced with TAK-242 pretreatment, whereas no significant change was observed in cells treated with TAK-242 alone (Figure 2C). These results further confirm that the proliferation of HO-8910PM cells is facilitated through the TLR4 signal activated by LPS.

Anchorage-independent growth is the ability of cancer cells to grow independently of a substrate, which reflects the proliferation potential of individual cells. Soft agar colony formation test was performed to measure the effect of TLR4 on anchorage-independent growth potential of HO-8910PM cells, and the results were similar to that of cell proliferation activity. The ability of tumor cells to form clones in soft agar was significantly enhanced after LPS stimulation, compared to non-LPS-stimulated cells. The clone formation rate was significantly decreased by TAK-242 treatment, compared to LPS treated cells (Figure 2D–2E).

**Figure 3:**
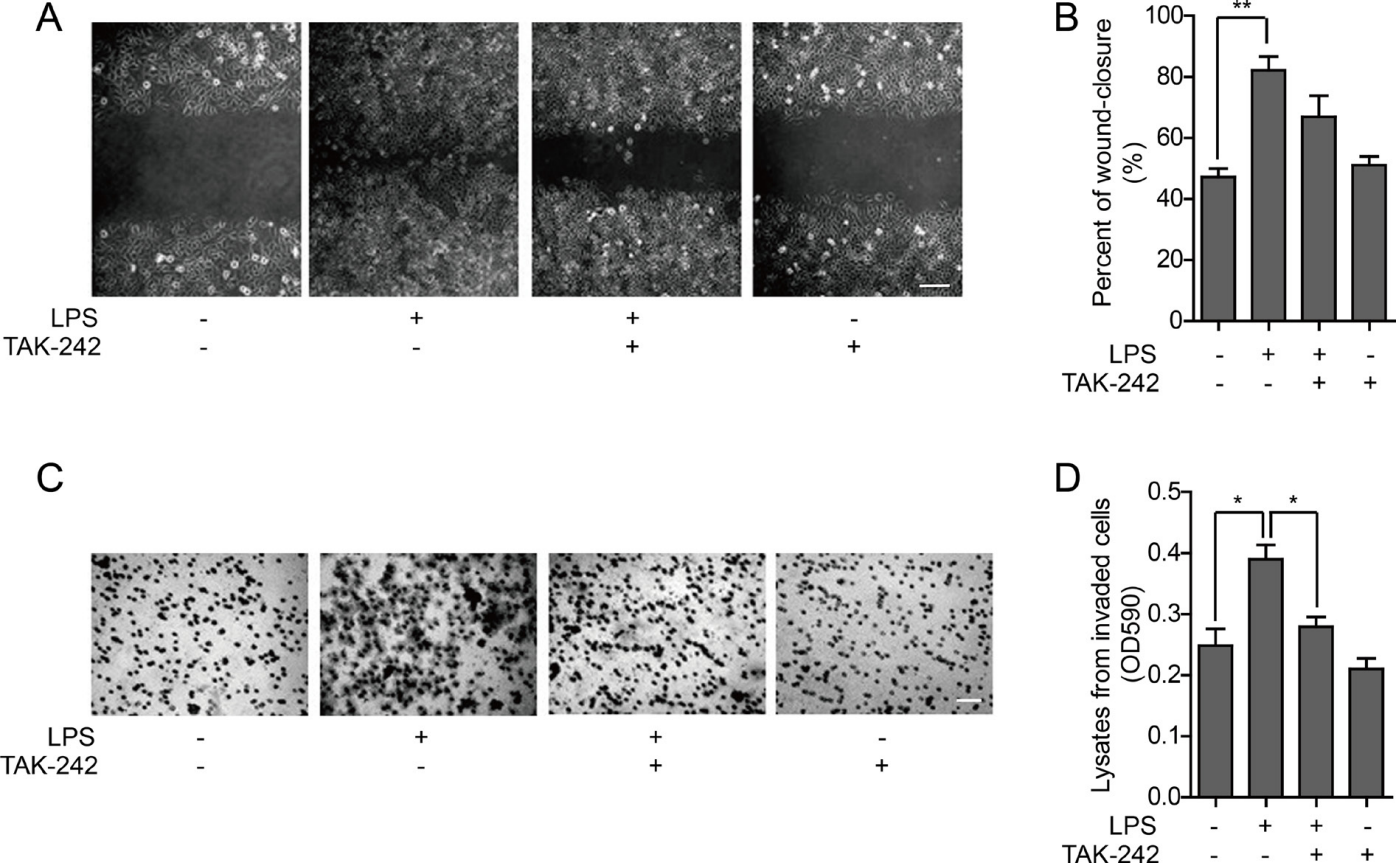
Effect of TLR4 on metastatic ability of HO-8910PM cells (**A**) Wound healing images of cells treated with LPS and/or TAK-242 at low magnification (Scale bar: 100 μm). (**B**) The wound-closure rates (%) were generated from the wound scale ratio as described in Materials and Methods. (**C**) Microscope images of cells treated with LPS and/or TAK-242 in cell invasion assay (Scale bar: 100 μm). (**D**) The cell invasion rates were evaluated as described in Materials and Methods. (A–D) Data are shown as mean ± SD of three independent experiments. ^*^P < 0.05; ^**^P < 0.01.

The wound healing assay can be used to investigate cell polarity and matrix reconstruction and evaluate cell migration ability. The wound healing rate of HO-8910PM cells, which is positively correlated with cell migration ability, was significantly increased by LPS stimulation. TAK-242 treatment did not significantly affect cell migration with or without LPS stimulation (Figure 3A–3B). A chemotaxis chamber enveloped with extracellular matrix was used to test the invasion activity of HO-8910PM cells treated with LPS and/or TAK-242. It was found that the cell invasion was significantly enhanced by LPS stimulation, whereas no significant change was observed in cells treated with TAK-242 alone. However, the increased invasion ability of the LPS-stimulated cells was significantly reduced after pretreatment with TAK-242 (Figure 3C–3D).

### Effect of OPN on proliferation and metastatic potential of ovarian cancer cells

**Figure 4:**
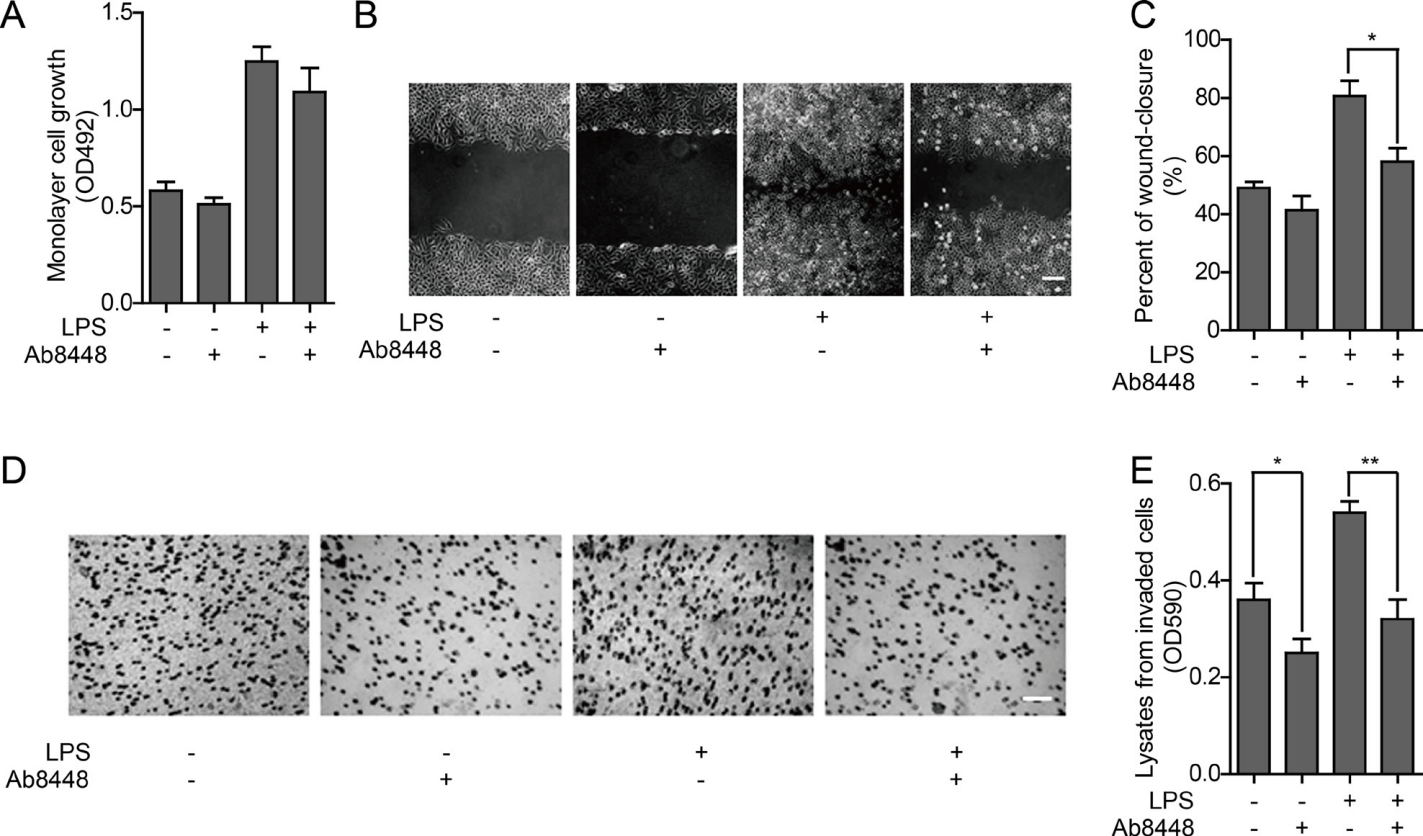
Effect of OPN on proliferation and metastatic ability of HO-8910PM cells (**A**) Cell proliferation ability was measured by MTT assay in cells with or without OPN neutralizing antibody Ab8448. (**B**) Inverted microscopic images of cells treated with LPS and/or Ab8448 in wound healing assay (Scale bar: 100 μm). (**C**) The wound-closure rates (%) were generated from the wound scale ratio as described in Materials and Methods. (**D**) Microscope images of cells treated with LPS and/or Ab8448 in cell invasion assay (Scale bar: 100 μm). (**E**) The cell invasion rates were evaluated as described in Materials and Methods. (A–E) Data are shown as mean ± SD of three independent experiments. ^*^P < 0.05; ^**^P < 0.01.

To determine whether secreted OPN affects cell proliferation, we performed an MTT assay after blocking the secreted OPN in HO-8910PM cells with an OPN neutralizing antibody, Ab8448. Proliferative activity was not significantly inhibited after treatment with the neutralizing antibody, although there was modest inhibition when the OPN antibody was added after LPS pretreatment (Figure 4A). In the wound healing assay, cell migration was enhanced following LPS stimulation, which could be inhibited by Ab8448 (Figure 4B–4C).

Given that the *in vitro* invasion activity of HO-8910PM cells was significantly enhanced by LPS stimulation, we questioned whether treatment with Ab8448 impairs this phenotype. We found that invasion activity of HO-8910PM cells with or without LPS stimulation could both be inhibited by blocking OPN with Ab8448 (Figure 4D–4E).

### Effect of TLR4 on OPN mediated metastatic phenotype of ovarian cancer cells

**Figure 5:**
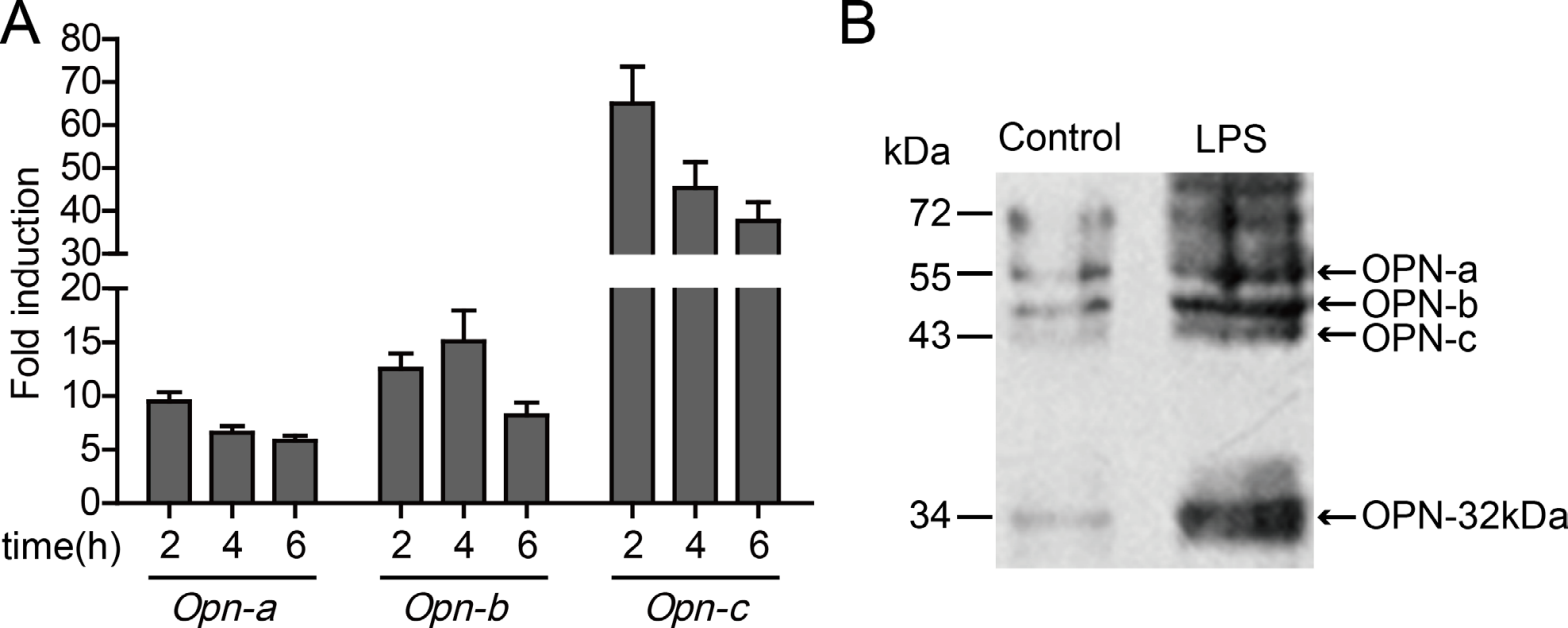
Effect of TLR4 on OPN expression in HO-8910PM cells (**A**) mRNA levels of OPN isoforms were analyzed by real-time PCR at indicated times after LPS stimulation. The amount of mRNA in untreated cells was given a value of 1.0. (**B**) The protein levels of OPN isoforms before and after LPS stimulation were measured by immunoblotting. (A–B) Data are shown as mean ± SD of three independent experiments.

Expression of OPN-a in HO-8910PM cells was rapidly up-regulated and peaked 2 hours after LPS stimulation. Compared with the non-stimulated group, the LPS-stimulated group exhibited a 10-fold up-regulation of OPN-a expression. Four hours after LPS stimulation, the expression of OPN-b was up-regulated 15-fold compared with that in the non-stimulated group. OPN-c expression was rapidly up-regulated 60-fold 2 hours after LPS stimulation. Subsequently, as the stimulation reduced and the degradation increased, mRNA levels of the three OPN isoforms decreased gradually (Figure 5A). To confirm whether the activation of TLR4 affected the OPN expression, the variation in the expression of secreted OPN in the supernatant of HO-8910PM cells before and after LPS stimulation was tested via immunoblotting. The protein levels of OPN-a, OPN-b, and OPN-c were all up-regulated at various extents after LPS stimulation, and the expression of OPN-32kDa was also significantly increased (Figure 5B). Overall, the OPN expression in ovarian cancer cells is significantly up-regulated by the activation of TLR4 via LPS stimulation.

**Figure 6:**
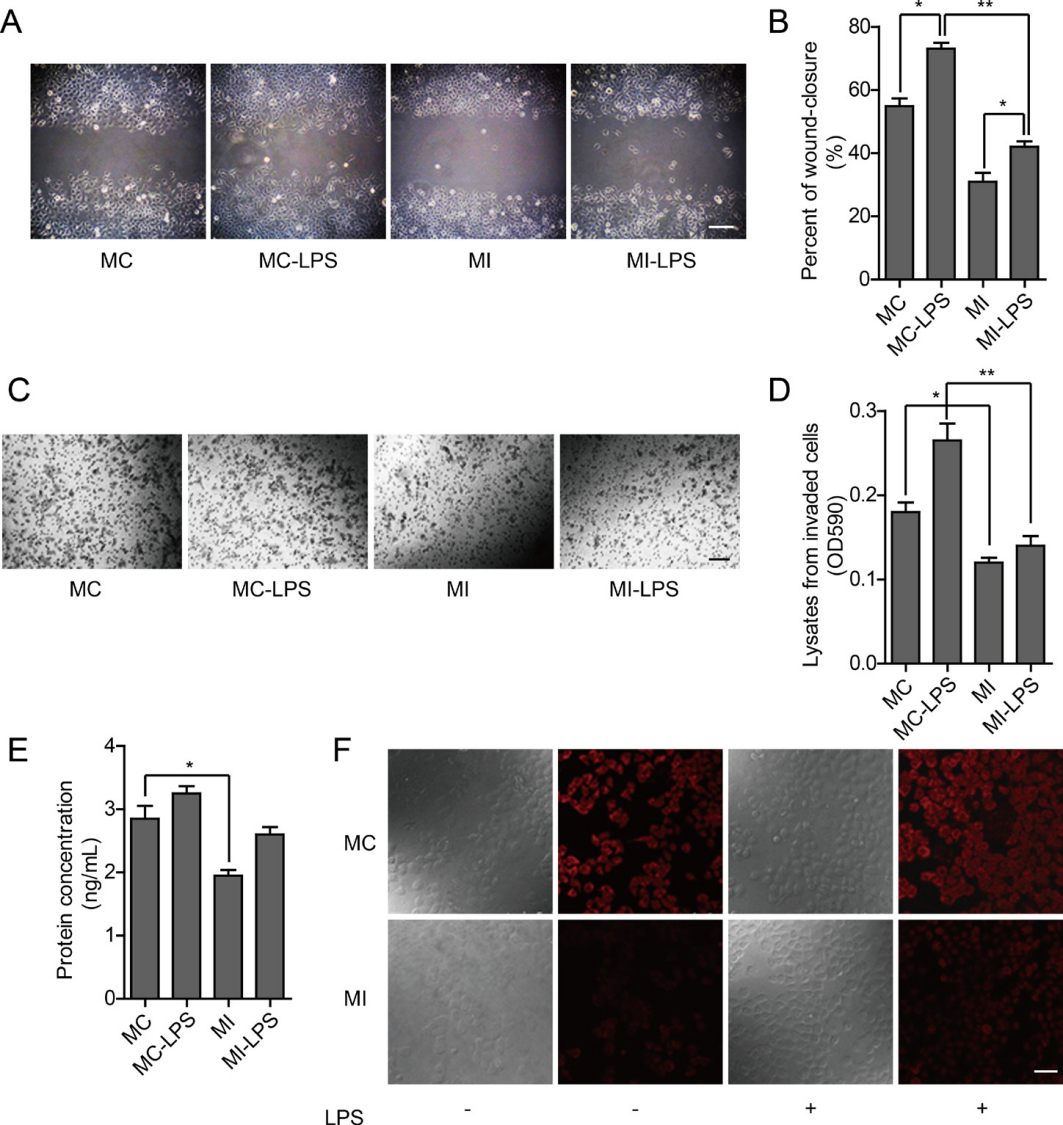
Effect of TLR4 in HO-8910PM cells with OPN knockdown (**A**) Inverted microscopic images of MC and MI cells in wound healing assay (Scale bar: 100 μm). (**B**) The wound-closure rates (%) were generated from the wound scale ratio as described in Materials and Methods. (**C**) Inverted microscopic images of MC and MI cells in cell invasion assay (Scale bar: 100 μm). (**D**) The cell invasion rates (%) were evaluated as described in Materials and Methods. (**E**) OPN protein levels in MC and MI cells were measured by ELISA according to the human OPN standard curve (not shown). (**F**) OPN expression (red) in MC and MI cells was analyzed by immunofluorescence. (A–F) MC: untreated HO-8910PM cells; MC-LPS: LPS-treated MC cells; MI: untreated HO-8910PM cells with OPN knockdown; MI-LPS: LPS-treated MI cells. Data are shown as mean ± SD of three independent experiments. ^*^P < 0.05; ^**^P < 0.01.

To explore the association between TLR4 signal pathway and OPN, as well as the possible mechanism that OPN promoted the metastasis phenotype of ovarian cancer cells, we used RNAi to knockdown OPN in HO-8910PM cells. In the wound healing assay and *in vitro* invasion assay of MC (HO-8910PM) and MI (HO-8910PM with OPN knockdown) cells, migration and invasion of MI cells was reduced compared to MC cells. Following LPS stimulation, migration and invasion of both MC and MI cells were significantly increased, but the extent of increase was reduced in MI cells compared to MC cells (Figure 6A–6D), suggesting that OPN is necessary for optimal LPS-stimulated migration and invasion. Thus, the metastatic ability of ovarian cancer cells is promoted by TLR4 activation via the up-regulation of OPN expression.

Using ELISA and immunofluorescence to measure expression of OPN, we found it was significantly less in MI cells than that in MC cells, which increased following LPS stimulation. However, the extent of increase in MI cells was less than that in MC cells (Figure 6E–6F). These results demonstrate that the proliferation and metastasis of ovarian cancer cells are facilitated by TLR4 via the up-regulation of OPN expression. Thus, the up-regulation of OPN expression by TLR4 signal may be an important factor that could enhance proliferation and metastasis ability of cancer cells.

## DISCUSSION

Several studies have shown that the expression of TLR4 is associated with ovarian cancer progression, treatment resistance, and poor prognosis [27–30]. In this study, we found a high expression level of TLR4 in the human ovarian cancer cell line (HO-8910PM), suggesting that TLR4 elicited essential biological functions and could be used as a potential target for the immune treatment of cancer. Therefore, the effect of TLR4 on the proliferation and metastasis of ovarian cancer cells was studied and it was found that the proliferation, anchorage-independent growth, migration and invasion abilities are significantly increased after stimulation with LPS. These effects are TLR4 specific as treatment with a TLR4 inhibitor abrogates this phenotype. Taken together, activation of TLR4 signal pathway can enhance the proliferation and metastatic ability of ovarian cancer cells, which directly promotes a malignant metastatic phenotype.

OPN has been reported overexpressed in various cancers, and is regarded as a novel cancer marker [31–34]. OPN can be produced via secretion by malignant tumor cells and promotes self-adhesion, invasion, metastasis, neovascularization and new tumor formation [35, 36]. Kang *et al*. found that OPN increased the invasion of lung cancer cells by triggering ROCK signaling mediated by the FAK/PI3K/AKT pathway [37]. Song *et al*. demonstrated that OPN derived from tumor microenvironment could promote metastasis of hepatocellular carcinoma [38]. Shevde *et al*. reported that OPN was an effector of critical steps in tumor progression and metastasis, particularly in facilitating bone metastasis of breast cancer [39]. Our research findings are consistent with previous studies. The OPN splicing isoforms OPN-a, OPN-b, OPN-c, and OPN-32kDa were highly expressed in HO-8910PM cells. LPS stimulation significantly improved the proliferation, migration and invasion abilities of tumor cells, but these effects were significantly reduced after the secreted OPN was neutralized. These results indicate that OPN boosts tumor cell proliferation, migration and invasion, and further suggest that the LPS-induced aggravation of metastatic phenotype is mediated by OPN. Therefore, OPN has considerable potential as a tumor metastatic gene for the diagnosis and treatment of tumors. However, the study on OPN is still premature. If OPN and its receptors can be further disrupted from gene initiation to signal transduction, and the tumor invasion pathway can be blocked, the extent of cancer malignancy will be effectively reduced in the future.

Moreover, we found that OPN expression was up-regulated through the activation of TLR4 in HO-8910PM cells, which exacerbated the metastatic phenotype. When OPN expression was knocked down, the cancer cell proliferation and metastasis induced by LPS was significantly reduced. Thus, up-regulation of OPN expression following TLR4 activation was an important factor that facilitated the malignant metastasis of ovarian cancer cells. However, the possible molecular mechanism that TLR4 regulates OPN remains unclear. TLR4 induces inflammatory gene expression and contributes to the formation of inflammatory microenvironments, which promotes cancer development and metastasis [40–42]. TLR4 leads to the activation of mitogen-activated protein kinase (MAPK) and nuclear factor-kappa B (NF-κB), resulting in the secretion of pro-inflammatory cytokines as well as the induction of matrix metalloproteinases (MMPs) [43]. Furthermore, several studies reported that OPN enhanced the migration and invasion of cancer cells by up-regulating MMP-2 and MMP-9 activities via NF-κB pathway [44–47]. In this context, it has been indicated that NF-κB may be a key factor between TLR4 signaling and OPN/MMP axis. Further research should be conducted to clarify this mechanism.

In conclusion, osteopontin, induced by LPS stimulation, enhances proliferation and metastasis of ovarian cancer cells. Our study provides a theoretical basis for the mechanisms of tumorigenesis, development and metastasis, and TLR4 and OPN may serve as potential combined targets for future therapy of ovarian cancer.

## MATERIALS AND METHODS

### Cell culture

The human ovarian cancer cell line HO-8910PM was purchased from the Institute of Biochemistry and Cell Biology (Shanghai, China). Cells were cultured in RPMI-1640 medium (Invitrogen, Shanghai, China), supplemented with 10% fetal bovine serum (Gibco, Carlsbad, CA, USA), 100 U/ml Penicillin (Invitrogen) and 100 U/ml Streptomycin (Invitrogen). Cells were maintained at 37°C in a humidified atmosphere of 5% CO_2_.

### RNA isolation and reverse transcription-PCR

HO-8910PM cells in logarithmic phase were seeded at 2.0 × 10^5^ cells/ml. Total RNA was isolated using RNAiso Plus kit (TakaRa, Dalian, China) according to the manufacturer's instructions. The concentration was adjusted to 1 μg/μl.

Reverse transcription was done by M-MLV Reverse Transcriptase system (Promega, Madison, WI, USA) according to the manufacturer's instructions, and PCR was performed using β-actin as an internal control. The primers were designed by Primer Premier 5.0 software (Table 1). A series of pre-experiments under the same conditions with gradient cycle numbers were performed to confirm the plateau phase, then the optimal cycle number just lower than the number of plateau phase was chosen as the exponential phase. PCR settings were the following: denaturation at 94°C for 3 min, followed by 30 cycles of 94°C for 30 s, annealing at 55–60°C for 30 s, and 72°C for 90 s, with a final extension step of 72°C for 7 min. The relative quantities of mRNA were analyzed by Band Scan 5.0 software.

**Table 1:** Primers used for RT-PCR

Genes name	ACC. NO.	Primer sequences		Amplification length(bp)
		Forward (5′ to 3′)	Backward(5′ to 3′)	
TLR1	NM_003263.3	TTTGAAAATTGTGGGCACCTTACTG	AAGCAACATTGAGTTCTTGCAAAGC	337
TLR2	NM_003264.3	TGTGAACCTCCAGGCTCTG	GTCCATATTTCCCACTCTCAGG	256
TLR3	NM_003256.1	AGCCGCCAACTTCACAAG	AGCTCTTGGAGATTTTCCAGC	327
TLR4	NM_138554.3	ACAGAAGCTGGTGGCTGTG	TCTTTAAATGCACCTGGTTGG	291
TLR5	NM_003268.5	CATGACCATCCTCACAGTCACAAAG	GGGCATAACTGAAGGCTTCAAGG	365
TLR6	NM_006068.3	CATGACGAAGGATATGCCTTCTTTG	TATTGACCTCATCTTCTGGCAGCTC	382
TLR7	NM_016562.3	TTGGCTTCTGCTCAAATGC	CTAAAGGTTGGAATTCACTGCC	300
TLR8	NM_138636.4	GTCGACTACAGGAAGTTCCCC	GGGTAACTGGTTGTCTTCAAGC	260
TLR9	NM_017442.2	GTGCCCCACTTCTCCATG	GGCACAGTCATGATGTTGTTG	260
OPN-a	NM_001040058.1	CCTAGCCCCACAGAATGC	GGGACAACTGGAGTGAAAAC	291
OPN-b	NM_000582.2	CCTAGCCCCACAGACCCTT	GGGGACAACTGGAGTGAAAAC	250
OPN-c	NM_001040060.1	GAAAAGCAGAATGCTGTGTCCT	CAGAGTCGTTCGAGTCAATGG	160
β-actin	NM_001101.3	GCCGTCTTCCCTCCATCGTG	GGAGCCACACGCAGCTCATTGTAGA	208

### MTT assay

HO-8910PM cells were seeded in 96-well plates (2.0×10^4^ cells/well). After 12 h of incubation, experimental group was stimulated with 0.1 μg/ml LPS (Sigma, Santa Clara, CA, USA) dissolved in Phosphate Buffered Saline (PBS), while LPS was replaced by serum-containing medium in control group. The proliferation assay was done with 3 - (4, 5 – dimethylthiazol – 2 - ylx) - 2, 5 - diphenyltetrazolium bromide (MTT). 200 μl of 0.5 mg/ml MTT was added to the medium, and the cells were incubated at 37°C for 4 h. Then the culture medium was discarded and 150 μl DMSO (Invitrogen) was added to each well to dissolve the precipitate. The OD at 492 nm was measured at indicated time periods (0 h, 2 h, 4 h, 6 h, 8 h, 12 h) after stimulation using the Microplate Reader (Rayto, Shenzhen, China).

In seperate experiments, cells were divided into four groups: the control group (C) and LPS group (L) were pretreated with serum-containing medium, while the inhibitor group (I) and inhibitor+LPS group (I+L) were pretreated with 1 μg/ml TLR4 inhibitor TAK-242 (Invitrogen) instead. After 6 h of pretreatment at 37°C, culture medium was discarded in all groups. Then serum-free medium was added to C and I group, LPS (Sigma) was added to L and I+L group. All the groups were maintained in culture for another 6 h. MTT assay was performed as described above.

### Colony formation in soft agar

In colony formation assay, the 35 mm dishes were coated with 1.5 ml of base layer containing 0.5% of agar prepared with serum-containing medium. HO-8910PM cells in logarithmic phase were grouped and treated as described in MTT assay. Subconfluent cells were trypsinized and counted, adjusting to 1.0 × 10^4^ cells/ml, and then resuspended in serum-containing medium plus 20% fetal bovine serum (Gibco) and 0.7% agar. 3 ml of the agar-cell mixture was plated over the 1.5 ml base layer to make the top agar. After 14 days of incubation in growth medium, colonies were stained with crystal violet and photographed under inverted light microscope (Olympus, Tokyo, Japan). Total colonies per dish were counted using ImageJ software.

### Wound healing assay

HO-8910PM cells were seeded in 35 mm dishes at 2.0 × 10^5^ cells/ml and allowed to grow until confluence. The same grouping and pretreatment was performed as described above. After 24 h of incubation with mitomycin, the cell monolayer was scratched with a pipette tip (200 μl) to create a narrow wound-like gap. Shortly after wounding, the cells were washed with serum-free medium and incubated with serum-containing medium. The dishes were photographed at 0 h, 6 h, 12 h, 24 h, 48 h and 72 h using an inverted light microscope (Olympus). The wound spacing is measured using ImageJ software, and the wound-closure rate (%) was calculated using the following formula: Wound-closure rate % = 1- S_treated_ / S_control_ × 100%, where S_treated_ and S_control_ were the average wound spacing of three independent experiments from treated and control groups, respectively.

### Cell invasion assay

*In vitro* invasiveness was assayed using 24-well transwell chambers with polycarbonate membrane (Corning, New York, NY, USA) coated by 100 μl matrigel (BD, San Jose, CA, USA). Matrigel was diluted three times with cold serum-free PRMI-1640 medium, applied to the membrane, cultured at least 4–5 hours at 37°C, and rehydrated with serum-free medium. HO-8910PM cells were grouped and pretreated as above. 100 μl of cell suspension (5.0 × 10^5^ cells/ml) was seeded to the upper chamber, and the lower chamber was filled with 0.6 ml culture medium plus 10% FBS. After incubation at 37°C for 24 h, matrigel and cells that had not invaded were wiped off the upper surface of membrane with a cotton swab. The upper chambers were removed and inverted, drying in the air. Cells on the lower surface of membrane were stained with 500 μl of 0.1% crystal violet and examined microscopically. Four randomly chosen fields were photographed and counted to determine the cell numbers that had invaded through the membrane. Furthermore, the membranes were put into other 24-well plates containing 500 μl of 33% acetic acid. After 10 min of oscillation and dissolution, the OD of lysates from invaded cells was measured at 570 nm using the Microplate Reader (Rayto).

### OPN neutralizing assay

HO-8910PM cells were pretreated with LPS (0.1 μg/ml) for 6 h, and seeded in 96-well plates (2.0 × 10^4^ cells/well). After 7 h of incubation, OPN neutralizing antibody ab8448 (Abcam, Cambridge, MA, USA) was added to the culture medium with a final concentration of 200 μg/ml. Cells were incubated in culture for another 12 h, and then MTT assay, colony formation in soft agar, wound healing assay and cell invasion assay were performed under the same conditions as previously described.

### Immunoblotting

HO-8910PM cells in logarithmic phase were lysed with 200 μl lysis buffer [50 mM Tris-HCl (pH 7.5), 150 mM NaCl, 1% Tween-20, 0.2% NP-40, 10% glycerol, 10 mM NaF and 1 mM Na_3_VO_4_ supplemented with protease inhibitor cocktail]. Total protein concentration was measured using Coomassie brilliant blue staining. 40 μg of protein was loaded onto a 10% SDS-polyacrylamide gel, separated by electrophoresis and electroblotted onto a polyvinylidene difluoride (PVDF) membrane (Osmonics, Pittsburgh, PA, USA). The membranes were blocked with 5% defatted dry milk (Roche, Basel, Switzerland) for 2 h at 37°C and probed with rabbit anti-OPN antibody (1:4 000 dilution, Abcam) for 2 h at room temperature. After washing with TBST (20 mM Tris-HCl, 140 mM NaCl, 0.1% Tween-20), the membranes were subsequently incubated with the secondary antibody (1:8 000 dilution, Beyotime, Haimen, China) conjugated with horseradish peroxidase for another 1 h at room temperature. The immunoreactivity was visualized by enhanced chemiluminescence (ECL) system (Beyotime) and exposed to an X-ray film.

### Real-time PCR

Real-time PCR of TLR4 and OPN was performed using SYBR Green RealMasterMix (Invitrogen) according to the manufacturer's instructions, with a Rotor-Gene Q Real-time PCR instrument (Qiagen, Hilden, Germany). The primers used in this study were shown in Table 2, using β-actin as an internal control. The amplification scheme was: incubation for 5 min at 95°C, followed by 40 cycles of 10 s at 95°C, 15 s at 59°C and 20 s at 72°C, with a final extension step of 7 min at 72°C. Data were normalized to β-actin expression in each sample. In all cases, each PCR was performed with triplicate samples. Relative expression of mRNA was determined using the 2^(-ΔΔCt)^ method.

**Table 2:** Primers used for real-time PCR

Genes name	ACC. NO.	Primer sequences		Amplification length(bp)
		Forward (5′ to 3′)	Backward(5′ to 3′)	
TLR4	NM_138554.3	AAGATTACCAGCCGCCAA	GTAGATGACAAGCCATTATGAGAC	255
OPN-a	NM_001040058.1	CCTAGCCCCACAGAATGC	GGGACAACTGGAGTGAAAAC	291
OPN-b	NM_000582.2	CCTAGCCCCACAGACCCTT	GGGGACAACTGGAGTGAAAAC	250
OPN-c	NM-001040060.1	GAAAAGCAGAATGCTGTGTCCT	CAGAGTCGTTCGAGTCAATGG	160
β-actin	NM_001101.3	CCTGTACGCCAACACAGTGC	ATACTCCTGCTTGCTGATCC	211

### RNA Interfering and transfection

HO-8910PM cells were cultured at 5.0 × 10^5^ cells/ml in RPMI-1640 medium containing 10% FBS until 40% to 70% confluent. Then cells were transfected with GFP expressing plasmids pGPU6/GFP/Neo containing shRNA specifically targeting OPN or negative control shRNA, which were pre-designed with the following sequences: OPN sense, 5′-CACCGCAGCTTTACAACAAA TACTTCAAGAGAGTATTTGTTGTAAAGCTGCTTTT TTG-3′ and antisense, 3′-CGTCGAAA TGTTGTTTATGAAGTTCTCTCATAAACAACATTTCG ACGAAAAAACCTAG-5′; negative control sense, 5′-CACCGTTCTCCGAACGTGTCACGTCAAGAGA TTACGTGACACGTTCGGAGAATTTTTTG-3′ and antisense, 3′-CAAGAGGCTTGCCTCTTAAAAAA CCAG-5′. shRNA and plasmids were obtained from GenePharma (Shanghai, China). Cell transfection was carried out with Lipofectamine 2 000 reagent (Invitrogen) in accordance with the manufacturer's instructions. After transfection, HO-8910PM cells were used for MTT assay, wound healing assay and cell invasion assay as described above.

### ELISA

Transfected cells were plated in 35 mm dishes at 1.0 × 10^6^ cells/dish in 1 mL of serum-free medium and treated with or without LPS. The supernatants were collected, and enzyme-linked immunosorbent assay (ELISA) was used to measure OPN expression levels according to the manufacturer's protocols of OPN ELISA kits (Invitrogen).

### Immunofluorescence

Transfected cells grown on glass coverslips were fixed for 30 min in 4% paraformaldehyde and permeabilized with 0.5% Triton X-100 (Promega) in PBS buffered saline for 30 min. After washing with TBS-0.1% Triton X-100 (TBSTx), nonspecific binding sites were blocked with TBSTx-5% BSA (Roche) for 60 min. Then cells were applied sequentially with a 1:100 dilution of rabbit polyclonal antibody anti-OPN (Abcam) at 4°C overnight and a 1:200 dilution of Cy3-conjugated goat anti-rabbit IgG (Beyotime) at room temperature for 60 min in the dark. For negative control, PBS was added instead of primary antibody. Further, immunofluorescence was visualized under a laser-scanning confocal microscope (Zeiss, Oberkochen, Germany).

### Statistical analysis

All data in different experiments were processed by SPSS 13.0 and expressed as mean ± SD. The data shown were obtained from at least 3 independent experiments. Differences between data groups were evaluated for significance using Student *t*-test. Statistical significance was represented as ^*^P < 0.05, ^**^P < 0.01, ^***^P < 0.001.
